# Genome-wide identification and characterization of NBS-encoding genes in *Raphanus sativus* L. and their roles related to *Fusarium oxysporum* resistance

**DOI:** 10.1186/s12870-020-02803-8

**Published:** 2021-01-18

**Authors:** Yinbo Ma, Sushil Satish Chhapekar, Lu Lu, Sangheon Oh, Sonam Singh, Chang Soo Kim, Seungho Kim, Gyung Ja Choi, Yong Pyo Lim, Su Ryun Choi

**Affiliations:** 1 Molecular Genetics and Genomics Laboratory, Department of Horticulture, College of Agriculture and Life Science, Chungnam National University, Daejeon, 34134 Republic of Korea; 2 Department of Crop Science, College of Agricultural and Life Sciences, Chungnam National University, Daejeon, 34134 Republic of Korea; 3 Neo Seed Co., 256-45 Jingeonjung-gil, Gongdo-eup, Anseong, Gyeonggi Province 17565 Republic of Korea; 4 Center for Eco-friendly New Materials, Korea Research Institute of Chemical Technology, Daejeon, 34114 Republic of Korea

**Keywords:** *Raphanus sativus* L., NBS-encoding gene, Gene duplication, Evolution, Synteny, *Fusarium oxysporum*

## Abstract

**Background:**

The nucleotide-binding site–leucine-rich repeat (NBS-LRR) genes are important for plant development and disease resistance. Although genome-wide studies of NBS-encoding genes have been performed in several species, the evolution, structure, expression, and function of these genes remain unknown in radish (*Raphanus sativus* L.). A recently released draft *R. sativus* L. reference genome has facilitated the genome-wide identification and characterization of NBS-encoding genes in radish.

**Results:**

A total of 225 NBS-encoding genes were identified in the radish genome based on the essential NB-ARC domain through HMM search and Pfam database, with 202 mapped onto nine chromosomes and the remaining 23 localized on different scaffolds. According to a gene structure analysis, we identified 99 NBS-LRR-type genes and 126 partial NBS-encoding genes. Additionally, 80 and 19 genes respectively encoded an N-terminal Toll/interleukin-like domain and a coiled-coil domain. Furthermore, 72% of the 202 NBS-encoding genes were grouped in 48 clusters distributed in 24 crucifer blocks on chromosomes. The U block on chromosomes R02, R04, and R08 had the most NBS-encoding genes (48), followed by the R (24), D (23), E (23), and F (17) blocks. These clusters were mostly homogeneous, containing NBS-encoding genes derived from a recent common ancestor. Tandem (15 events) and segmental (20 events) duplications were revealed in the NBS family. Comparative evolutionary analyses of orthologous genes among *Arabidopsis thaliana*, *Brassica rapa*, and *Brassica oleracea* reflected the importance of the NBS-LRR gene family during evolution. Moreover, examinations of cis-elements identified 70 major elements involved in responses to methyl jasmonate, abscisic acid, auxin, and salicylic acid. According to RNA-seq expression analyses, 75 NBS-encoding genes contributed to the resistance of radish to Fusarium wilt. A quantitative real-time PCR analysis revealed that *RsTNL03* (*Rs093020*) and *RsTNL09* (*Rs042580*) expression positively regulates radish resistance to *Fusarium oxysporum*, in contrast to the negative regulatory role for *RsTNL06* (*Rs053740*).

**Conclusions:**

The NBS-encoding gene structures, tandem and segmental duplications, synteny, and expression profiles in radish were elucidated for the first time and compared with those of other Brassicaceae family members (*A. thaliana*, *B. oleracea*, and *B. rapa*) to clarify the evolution of the NBS gene family. These results may be useful for functionally characterizing NBS-encoding genes in radish.

**Supplementary Information:**

The online version contains supplementary material available at 10.1186/s12870-020-02803-8.

## Background

Plants contain numerous resistance (R) genes that are vital for immunity against viral, fungal, and bacterial pathogens [1–3]. Specifically, R gene-mediated disease resistance is one of the most important plant mechanisms related to defense against pathogens [1]. The R genes are grouped in the subsequent five functionally diverse category based on respective domains: first, nucleotide-binding site–leucine-rich repeat (NBS-LRR) genes, sub-grouped as Toll/interleukin 1 receptor (TIR)-NBS-LRR (TNL) and coiled-coil (CC)-NBS-LRR (CNL) genes; second, receptor-like transmembrane proteins; third, serine–threonine kinases; fourth, receptor-like kinases (RLKs); and fifth, atypical R genes [4]. The predominant class of R genes includes genes with NBS and LRR domains [1, 5, 6]. To date, in all plant species over 300 R genes have been detected and cloned, of which more than 60% encode NBS and LRR domains [7]. The three NBS-LRR subclasses are TNL, CNL, and resistance to powdery mildew 8 (RPW8)-NBS-LRR (RNL), which are distinguished based on the differences in the N-terminal domains in angiosperms [8]. The TNL and CNL proteins are mainly responsible for the recognition of specific pathogens, whereas RNL proteins participate in downstream defense signal transduction pathways [9]. Therefore, Bonardi et al. described RNL proteins as helper NBS-LRRs [10].

The LRR domain, which is located at the C-terminal of plant NBS-LRR proteins, comprises tandem LRRs involved in the detection of invading pathogens [11]. The NBS domain is a functional ATPase domain, with its nucleotide-binding state believed to regulate R protein activities and function as a molecular switch [12, 13]. Although the TIR and CC domains are implicated in signaling and resistance specificity, their associated pathways differ. The TIR domain is mostly involved in self-association and homotypic interactions with other TIR domains [14, 15], whereas the CC domain may be related to protein–protein interactions and signaling [16]. To protect against diverse and rapidly evolving pathogens, a single plant genome usually encodes hundreds of NBS-LRR genes. The data generated in recent whole-genome sequencing analyses have enabled researchers to comprehensively analyze NBS-LRR genes in economically important plants [17–20].

Radish (*Raphanus sativus* L.), which is one of the more prominent members of the family *Brassicaceae*, is an economically valuable root vegetable crop grown worldwide [21]. Radish quality and yield are influenced by biotic stresses, including fungal and bacterial diseases as well as infestations by insect pests [22]. Specifically, Fusarium wilt caused by the soil-borne fungal pathogen *Fusarium oxysporum*, can severely damage radish and numerous other types of vegetables [23]. Because of its broad host range, *F. oxysporum* can survive at relatively high soil temperatures (> 24 °C) and remain viable even in the absence of any host plant, making it difficult to control [24, 25]. In addition to the TNL-type resistance genes *FOC1* and *FocBo1*, which are responsible for the resistance of *Brassica oleracea* to *F. oxysporum*, many NBS-LRR genes related to Fusarium wilt resistance have been recently identified in diverse plant species [26–28].

Other genes belonging to the NBS-LRR family have been detected in various plants, including *Arabidopsis thaliana* [17, 29], *Brassica rapa* [30], chickpea [31], and *Gossypium* species [32]. Unfortunately, to the best of our knowledge, the potential roles of radish NBS-LRR genes related to disease resistance have not been investigated. The available radish genome sequence is a useful resource for the whole-genome identification of transcription factor families [33, 34]. However, the effects of *F. oxysporum* infections on radish NBS-LRR genes and their families remain unexplored. Therefore, combining bioinformatics and gene expression analyses to systematically study the evolution, expression, and potential functions of NBS-LRR genes may help to improve our understanding of the regulatory networks involved in radish plant growth and in response to *F. oxysporum*.

In this study, a genome-wide analysis of the radish genome identified 225 NBS-encoding genes (99 full NBS-LRR and 126 partial NBS genes) divided into two subclasses (CNL and TNL). These genes were further characterized regarding chromosomal locations, structures, and duplications. Additionally, with a focus on the NBS-LRR genes, we examined the encoded conserved domains as well as phylogenetic relationships and synteny with genes from *A. thaliana*, *B. rapa*, and *B. oleracea*. Furthermore, the pathogen-induced NBS-LRR gene expression profiles indicated that some R genes are differentially expressed in genetically different germplasm of radish (resistant and susceptible). Our results provide crucial insights into the evolution of this gene family in the radish genome. Moreover, the extensive R gene data presented herein may be useful for accelerating future breeding efforts aimed at improving the disease resistance of radish and other *Brassicaceae* crops.

## Results

### Identification and classification of NBS-encoding genes in *R. sativus*

**Fig. 1:**
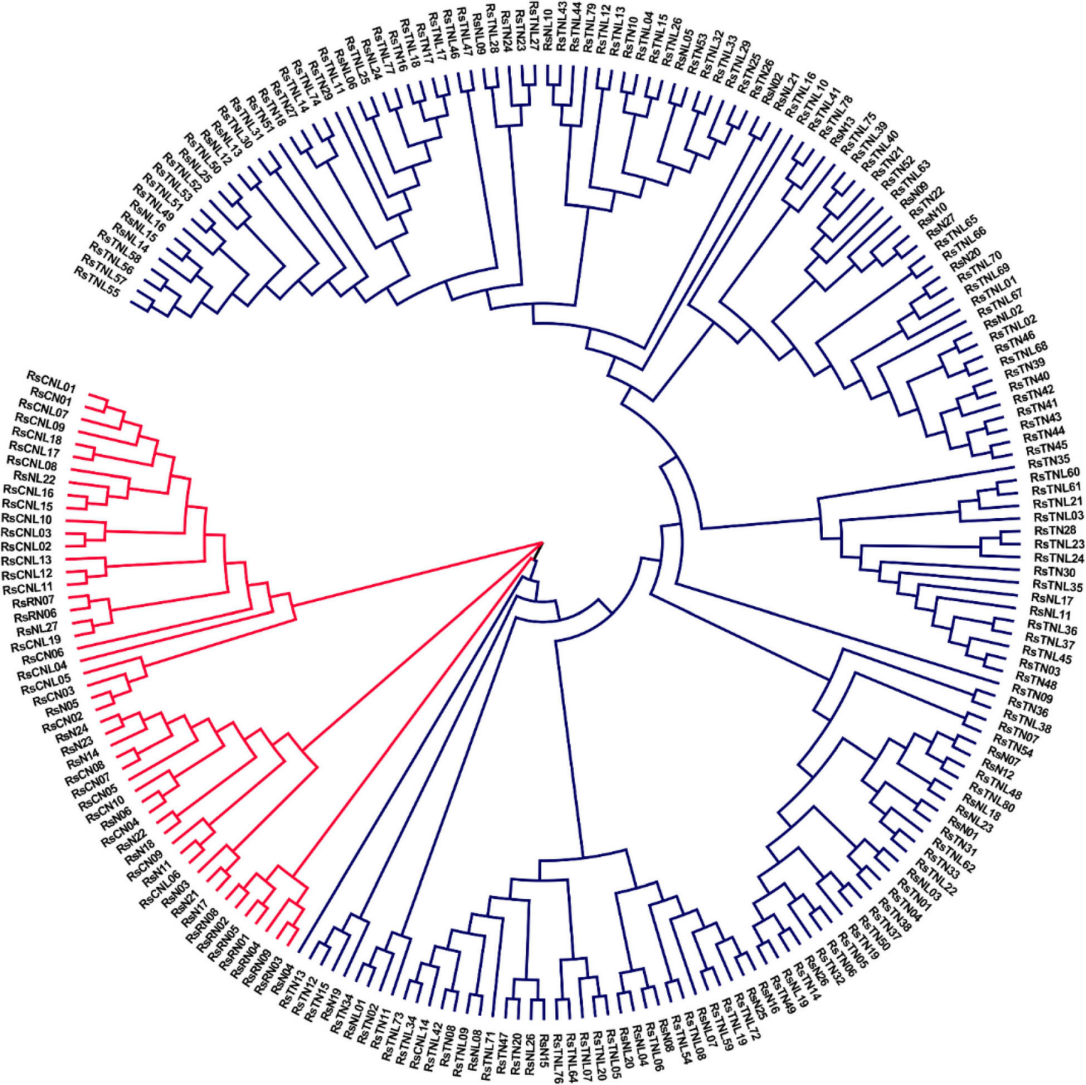
Phylogenetic analysis of the radish NBS-LRR proteins. A maximum likelihood phylogenetic tree was constructed based on 225 NBS-encoding genes. The bootstrap values (1000 iterations) are provided on each branch. The proteins are designated as follows: Rs + Domain (TNL, TN, NL, CNL, CN, RN, N, and NL). Red and blue correspond to CNL and TNL clades, respectively. The radish NBS-encoding gene ID and the protein sequence information are provided in Additional file 1: Table S1

To comprehensively identify potential NBS-encoding genes in radish, the hidden Markov model (HMM) profile NB-ARC (Pfam: PF00931) from the Pfam database was used to screen the protein sequences encoded in the radish genome [34]. A total of 488 gene candidates with an NBS-LRR domain were identified. These candidate NBS-encoding genes were manually screened and functionally annotated according to the closest *A. thaliana* homolog. Finally, 225 non-redundant NBS-encoding R gene candidates were identified in the Rs1.0 genome (Table 1, Additional file 1: Table S1). The NBS-containing candidate proteins were classified into the TNL or CNL subfamilies based on their HMM profiles and the NCBI Conserved Domain Database, with relationships visualized in a phylogenetic tree (Fig. 1, Table 1). The phylogenetic tree clearly distinguished the TNL and CNL genes in two separate clades (Fig. 1). Gene names were assigned based on their domain type and chromosomal position. The TNL subfamily included 80 genes with full-length domains (TIR, NBS, and LRR) as well as 54 TN (TIR and NBS, but no LRR), 25 NL_TNL_ (NBS and LRR, but no TIR), and 15 N_TNL_ (no TIR or LRR) genes. The remaining 51 genes belonging to the CNL subfamily included 19 with full-length domains (CC, NBS, and LRR) as well as 10 CN (CC and NBS, but no LRR), 9 RN (RPW8 and NBS, but no LRR), 2 NL_CNL_ (NBS and LRR, but no CC), and 11 N_CNL_ (no CC or LRR) genes. Genes encoding RPW8-NBS-LRR proteins were not detected in the radish genome. We also analyzed the well-characterized NBS-encoding genes from the genetically closely related plant species *A. thaliana*, *B. rapa*, and *B. oleracea* (Table 1) using the same method. A total of 164 (*A. thaliana*), 212 (*B. rapa*), and 244 (*B. oleracea*) NBS-encoding genes were identified.

**Table 1:** Summary of NBS gene in *R. sativus*, *A. thaliana*, *B. rapa* and *B. oleracea.*

Predicted Type	Letter code	*R.sativus*	*A.thaliana*	*B.rapa*	*B.oleracea*
**TNL type**					
TIR NB LRR	TNL	80	75	81	93
NB LRR	NL_TNL_	25	4	7	11
TIR NB	TN	54	26	38	50
NB	N_TNL_	15	2	5	23
**CNL type**					
CC NB LRR	CNL	19	18	19	15
RW8 NB LRR	RNL	0	1	1	1
NB LRR	NL_CNL_	2	3	15	3
CC NB	CN	10	11	15	16
RW8 NB	RN	9	4	6	12
NB	N_CNL_	11	20	25	20
Total		225	164	212	244

### Genomic distribution among radish chromosomes

**Fig. 2:**
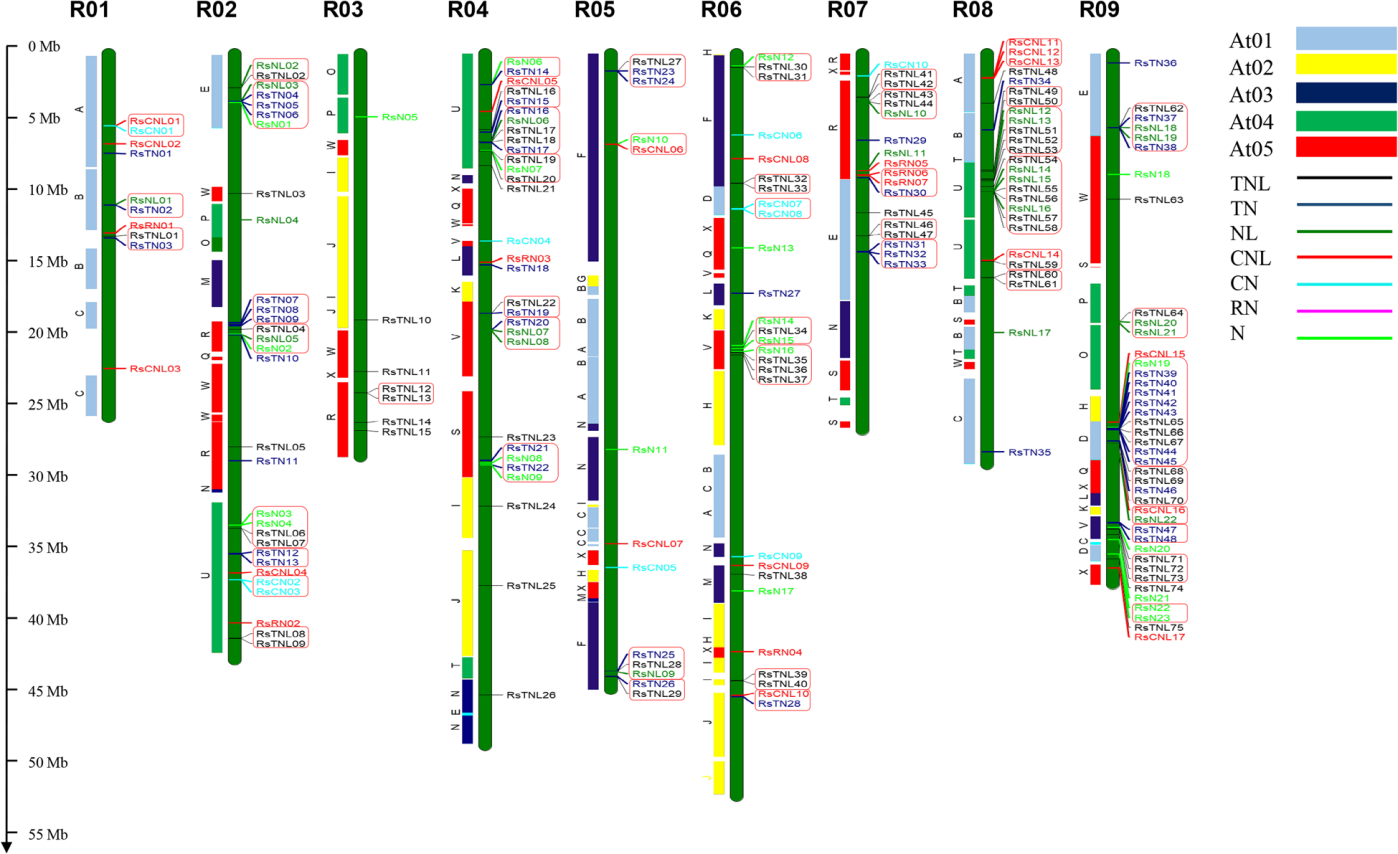
Chromosomal localization and clustering of NBS-encoding genes in the *R. sativus* genome. The distribution of 202 genes on nine chromosomes (R01–R09) is presented. Different NBS types are marked by different colors. Gene clusters are indicated by red rectangles. The left side of chromosomes represents the syntenic regions in *A. thaliana*. The *A. thaliana* chromosomes are designated as At01–05, which are presented in different colors. The conserved chromosomal blocks in crucifer genomes are indicated by A–X [35]

Of the 225 NBS-encoding genes, 202 were mapped onto nine radish chromosomes, whereas the other 23 genes were located on several scaffolds which were not mapped on the chromosome of *R. sativus* (Fig. 2, Additional file 1: Table S1). We determined the distribution of CNL, TNL, and partial NBS-encoding genes on different chromosomes (Additional file 2: Fig. S1). Moreover, the TNL genes were almost uniformly distributed on chromosomes R01 to R09, whereas the CNL genes were not detected on chromosomes R03 and R06. Furthermore, chromosome R09 had the most NBS-encoding genes (41), whereas chromosome R03 had the fewest (7). The ratio of radish TNL:CNL genes was almost 4:1 (80:19), which was consistent with the corresponding ratios in *A. thaliana* (78:18), *B. rapa* (74:20), and *B. oleracea* (91,20) [17].

There is no known pattern in the chromosomal distribution of NBS-encoding genes, with most of them detected in clusters. This distribution may facilitate sequence exchanges via recombination mispairing. We identified NBS-encoding gene clusters based on previously established criteria that an NBS-encoding cluster should have two or more genes separated by fewer than 200 kb, with no more than eight non-NBS-encoding genes in between [36]. On radish chromosomes, 146 (72%) NBS genes were mapped in 48 clusters, whereas the remaining 55 genes were detected as singletons (Fig. 2 and Additional file S2). Our analysis revealed that chromosome R09 has the most NBS genes (41; 20.30% of the mapped genes) distributed in eight clusters, in addition to nine singletons. Cluster sizes varied across the genome (2–11 genes). Cluster 44 was the largest, with 11 genes belonging to the TNL subfamily.

To clarify the evolutionary relationships among genes, we analyzed the distribution of NBS-LRR genes among the crucifer blocks in the radish genome. Of the 24 identified blocks (Fig. 2) [35], the U block on chromosomes R02, R04, and R08 was the largest (48 NBS-encoding genes), followed by the R block (24 genes), D block (23 genes), E block (23 genes), and F block (17 genes). The U block may be one of the most important in terms of NBS-encoding genes.

### Gene characteristics and structure

The lengths of the genomic and coding sequences of the 225 NBS-encoding genes as well as the length, molecular weight (MW), and isoelectric point (pI) of the corresponding proteins were comprehensively analyzed (Additional file 1: Table S1). The genomic and coding sequence lengths ranged from 336 bp (RsN02) to 11,267 bp (RsTNL06) and from 1149 bp (RsN02) to 4899 bp (RsTNL15), respectively. The protein lengths ranged from 111 amino acids (RsN02) to 1632 amino acids (RsTNL15). There were also significant variations in the MW and pI, which ranged from 12.72 kDa (RsN11) to 182.40 kDa (RsTNL15) and from 4.77 to 9.60, respectively. Additionally, the average MW of the TNL (122.27 kDa) and CNL (99.84 kDa) proteins were markedly different.

**Fig. 3:**
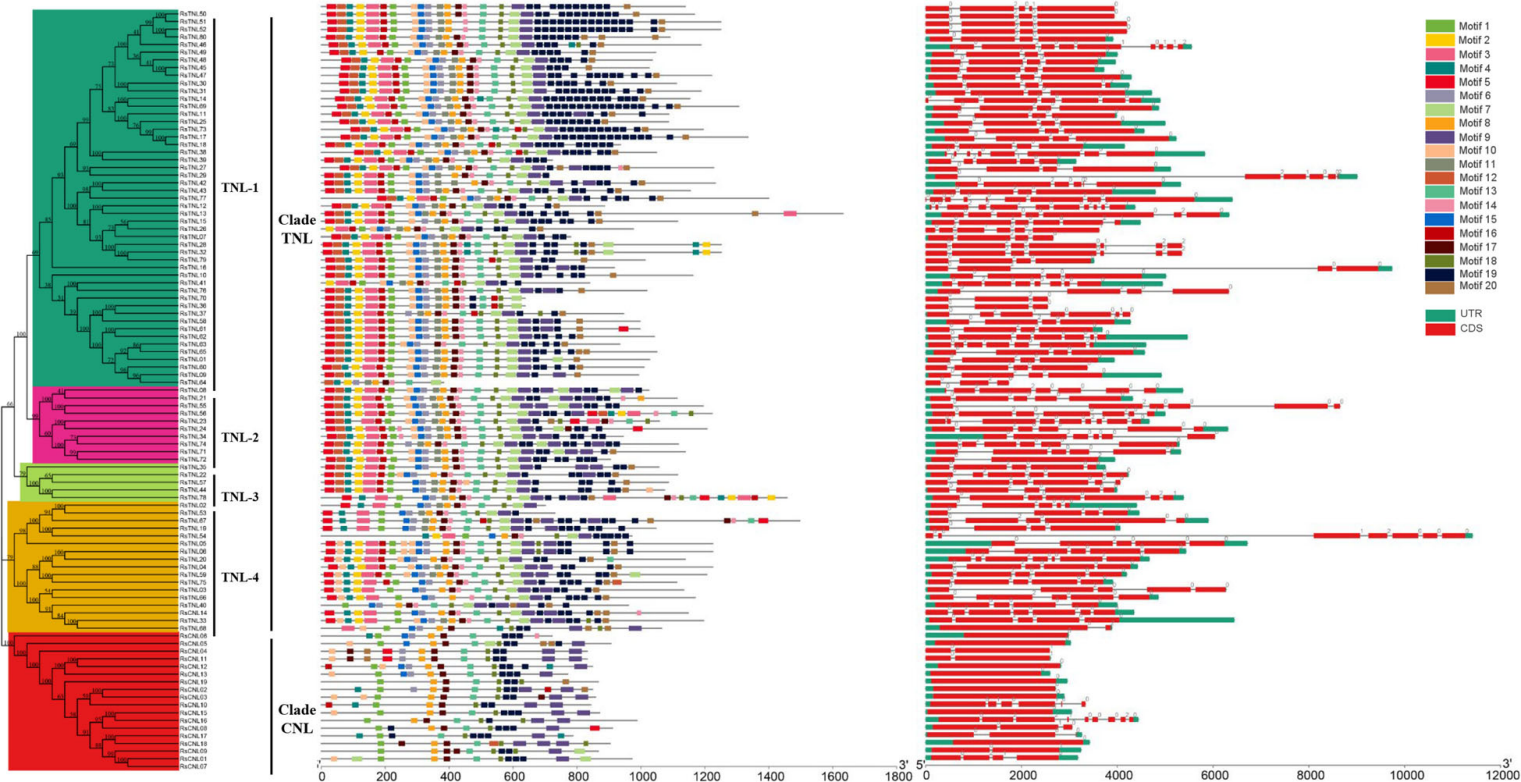
Circos diagram of segmentally duplicated NBS-encoding genes in the radish genome. Gray lines indicate the syntenic blocks in the radish genome. Red lines indicate the duplicated pairs of NBS-encoding genes. Chromosome numbers are provided on each chromosome

To assess the structural diversity of the *R. sativus* NBS-encoding genes, we compared the number of exons. The full-length CNL and TNL genes in the radish genome had an average of 2.42 and 5.26 exons, respectively (Fig. 3), which is consistent with the analyses of *A. thaliana*, *B. rapa*, and *B. oleracea*, in which the CNL and TNL genes have an average of 2.2, 2.3, and 3.4, and 5.3, 5.2, and 6.4 exons, respectively. Moreover, 47.37% of the CNL genes were encoded by a single exon. These results indicate that the number of introns may have increased and decreased during the structural evolution of the two types of NBS-LRR resistance genes in radish.

### Cis-element analysis

Cis-elements in promoters are usually involved in gene regulation. Thus, to further investigate the potential regulatory networks of the NBS-encoding genes, the cis-elements in the sequences 2 kb upstream of the start codon of 225 NBS-encoding genes were analyzed with PlantCARE (Additional file 1: Table S3). A total of 70 cis-elements were detected, including 10 hormone-responsive elements, 33 light-responsive elements, 4 elements related to abiotic stress, 8 elements associated with tissue-specific expression, and 15 other elements. The cis-element DRE, which is a common cis-acting element in promoter and enhancer regions, was detected in the promoter region of 218 NBS-encoding genes. Additionally, an AT-rich fragment, which is a core promoter element located approximately 30 bp upstream of the transcription start site, was detected in 216 NBS-encoding genes. This sequence was considered to be an essential element in the promoter of the NBS-encoding genes. The promoter region of 159 NBS-encoding genes included the CGTCA-motif, which is a cis-acting regulatory element associated with methyl jasmonate-responsiveness. The abscisic acid-responsive element influencing abscisic acid responsiveness was detected in the promoter region of 165 genes, suggesting that NBS-encoding genes are involved in plant responses to pathogen infections. Moreover, a TCA-element, which is involved in salicylic acid responses, was detected in 97 gene promoters, whereas the TGA-element related to auxin responses was identified in 82 gene promoters. Furthermore, the P-box and GARE-motif associated with gibberellin-responsiveness were present in 55 and 40 NBS-encoding gene promoters, respectively. These results may be relevant for developing a method for identifying candidate genes related to disease resistance.

### Conserved motifs and phylogenetic relationships among TNLs and CNLs

**Fig. 4:**
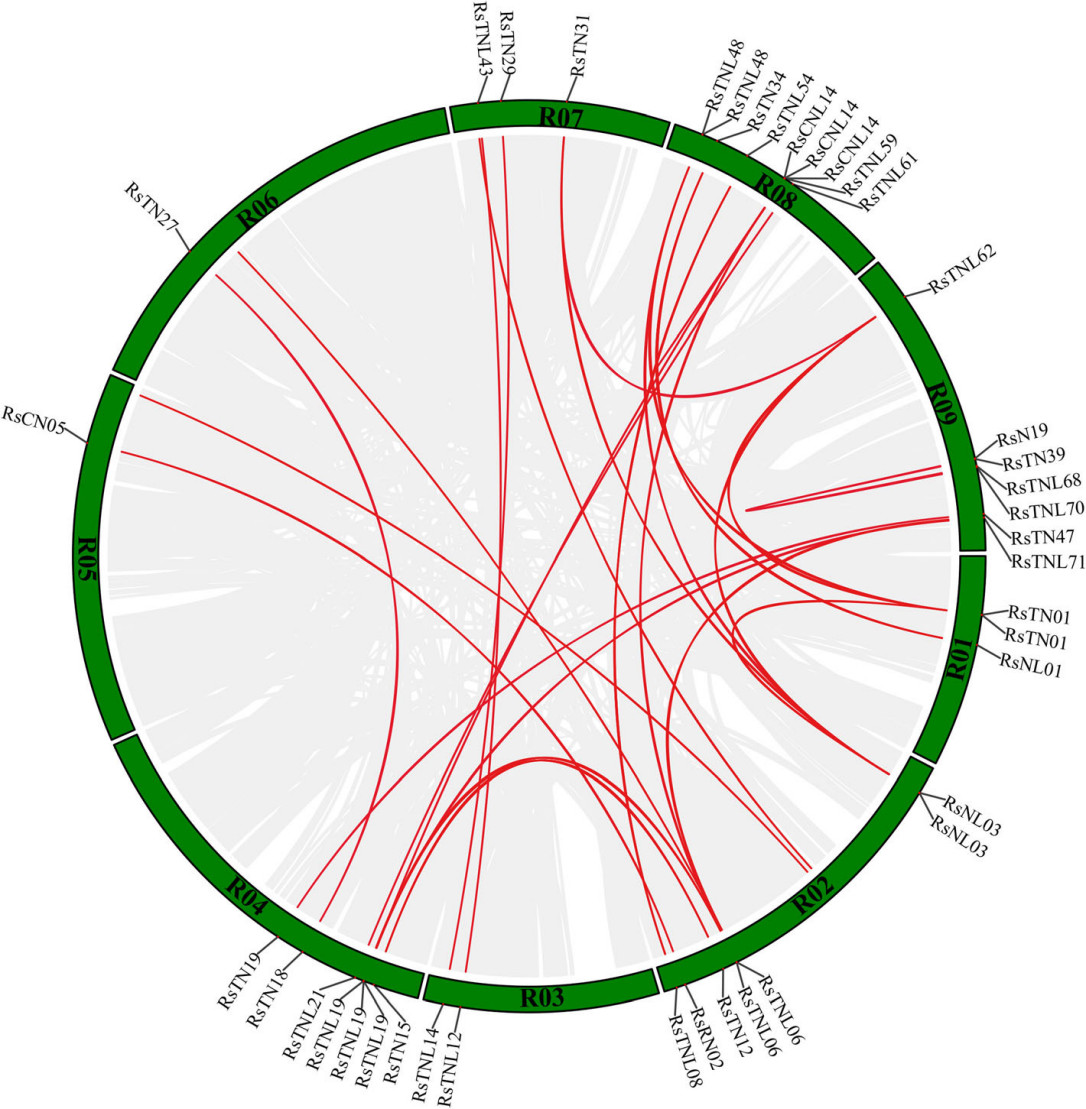
Phylogenetic relationships, structure, and the encoded conserved motifs of the CNL and TNL genes. **a** Phylogenetic tree constructed based on the full-length radish protein sequences with the MEGA-X program. Different clades are presented in different colors. **b** Motif compositions of radish CNL and TNL proteins. Motifs 1–20 are displayed in different colored boxes. The sequence information for each motif is provided in Additional file S5. **c** Exon–intron structures of radish CNL and TNL genes. Green boxes represent untranslated 5′ and 3′ regions. Red boxes and gray lines indicate exons and introns, respectively. Gene and protein lengths can be estimated with the scale at the bottom

To investigate the TNL and CNL gene and protein structures, we built a phylogenetic tree based on the full-length amino acid sequences encoded by the *R. sativus* TNL and CNL genes*.* The phylogenetic analysis indicated that the radish NBS-LRRs can be divided into two large groups, namely CNL and TNL (Fig. 4 and Additional file 1: Table S4). The RsTNL group comprised four subgroups. Of the 80 RsTIR proteins, 49, 10, 5, and 16 belonged to subgroups RsTNL-1, RsTNL-2, RsTNL-3, and RsTNL-4, respectively. These results were identical to those of phylogenetic analyses of *R. sativus* and *A. thaliana* (Additional file Fig. 2).

To further elucidate the potential functions and diversification of the TNL and CNL genes in *R. sativus*, 20 encoded conserved motifs were identified and numbered 1–20 based on the MEME program (Additional file 1: Tables S4 and S5). The TIR domain was detected in all RsTNLs. Additionally, the RsTNL-1 subgroup members had most of the motifs. The RsTNL-2 and RsTNL-3 proteins lacked motifs 20 and 11, respectively. The proteins in subgroup RsTNL-4 were missing motifs 11, 12, and 16 (Fig. 3). The RsCNL group members mostly had only six motifs, including the CC domain. Common motif compositions were revealed within subgroups. However, regarding the motif types and numbers, there was considerable diversity among subgroups. This suggests the proteins within subgroups are functionally similar.

### Tandem duplication and synteny analyses of NBS-encoding genes

Whole-genome and tandem duplications are critical events for enhancing genome complexity and evolutionary novelty. In the *R. sativus* genome, 34 of 225 NBS-encoding genes (15.11%) were associated with tandem duplications and were distributed in 15 tandem arrays of 2–5 genes (Additional file 1: Table S6). Our data also revealed variability in the number of duplicated genes per tandem duplication event and an uneven distribution of these duplications on five of nine chromosomes. Genes encoding domains (e.g., CNL, TNL, and TN) were present in the tandem arrays. The 14 tandem duplication events detected for chromosome R09 involved five *RsTN* genes. In contrast, single tandem duplication events occurred on chromosomes R02, R06, R07, R08, and R09, each involving two genes. Chromosome R08 had the most tandem arrays (six tandem groups containing 13 genes), reflecting a hot spot for the distribution of NBS-encoding genes. An analysis of our data according to BLASTP and MCScanX methods identified 20 segmental duplication events involving 32 NBS-encoding genes (Fig. 4 and Additional file 1: Table S7).

The Ka/Ks values (Additional file 1: Table S8) of the pairs of segmentally duplicated genes were less than 1, indicating these genes evolved under negative selection. These results suggest that both tandem and segmental duplication events were a major driving force for the evolutionary expansion of the NBS-encoding genes in the radish genome.

### Comparative synteny analyses of orthologous pairs of NBS-encoding genes

**Fig. 5:**
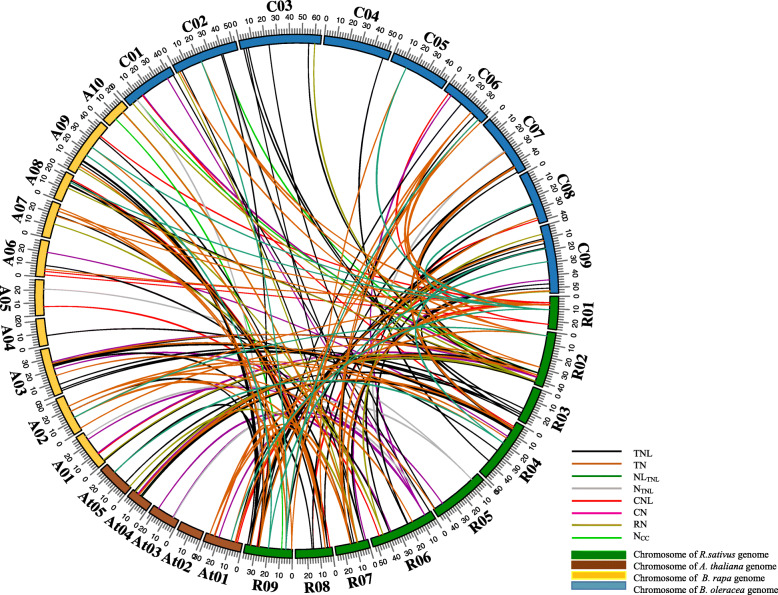
Syntenic relationships between radish NBS-encoding genes and *A. thaliana*, *B. rapa*, and *B. oleracea* genes. Green bars represent *R. sativus* chromosomes (R01–R09). Brown, yellow, and blue bars represent the chromosomes of *A. thaliana* (At01–At05), *B. rapa* (A01–A10), and *B. oleracea* (C01–C09), respectively

To further investigate the phylogenetic relationships among the radish NBS-encoding genes, we constructed three synteny maps comparing radish with *A. thaliana*, *B. rapa*, and *B. oleracea.* A total of 209 pairs of NBS-encoding genes (Additional file 1: Table S9) had syntenic relationships between *R. sativus* and *A. thaliana*, *B. rapa*, and *B. oleracea*. Specifically, 39 orthologous gene pairs were detected between *R. sativus* and *A. thaliana*, whereas there were 73 pairs between *R. sativus* and *B. rapa* as well as 97 pairs between *R. sativus* and *B. oleracea* (Fig. 5). Of the 39 pairs between *R. sativus* and *A. thaliana*, all were single-copy genes, except for *RsCN06*, which had two copies. Four partial NBS genes (*RsN*_*TIR*_*19*, *RsNL*_*TIR*_*07*, *RsTN31*, and *RsTN39*) in the radish genome were detected as homologous to *A. thaliana* TNL genes (*AT1G63730*, *AT5G45210*, and *AT1G63860*). The *A. thaliana* partial NBS gene *AT5G18350* corresponded to the complete NBS gene *RsTNL12*. Of the 73 gene pairs between *R. sativus* and *B. rapa*, 46 radish NBS-LRR genes were present as a single copy, whereas there were two copies of 12 genes and three copies of one gene (*RsTN01*). The *Bra027122* (no TIR domain) and *Bra030779* (no CC domain) *B. rapa* genes corresponded to *RsTNL75* and *RsCNL11*, respectively. Moreover, four *RsTN* genes (*RsTN26*, *34*, *39*, and *44*) and *RsNL01* were syntenic with TNL genes (*Bra001160*, *Bra024652*, *Bra027779*, and *Bra027772*), whereas *RsCN02* was syntenic with a CNL gene (*Bra019063*) in the *B. rapa* genome. Overall, we detected 59 radish NBS-encoding genes syntenic with 64 NBS-encoding genes in the *B. rapa* genome (Additional file 1: Table S9). There were about 97 homologous gene pairs between *R. sativus* and *B. oleracea*, among which 47, 19, and 4 NBS-encoding genes in radish were retained as one, two, and three copies, respectively, in *B. oleracea*, with the remaining genes lacking syntenic relationships. The partial NBS-encoding genes *RsTN11*, *RsTN31*, *RsNL07*, and *RsN19* were syntenic with complete TNL genes (*Bo2g010720*, *Bo8g104700*, *Bo9g061200*, and *Bo9g029350*) in the *B. rapa* genome. Additionally, two *RsCNL* genes (*RsCNL03* and *RsCNL05*) as well as four *RsTNL* genes (*RsTNL09*, *RsTNL14*, *RsTNL62*, and *RsTNL72*) corresponded to partial NBS genes (*Bo6g020950*, *Bo1g048080*, *Bo3g154220*, *Bo6g089230*, *Bo2g126980*, and *Bo3g006960*) in the *B. rapa* genome. We detected a total of 70 radish NBS-encoding genes syntenic with 67 NBS-encoding genes in the *B. oleracea* genome (Additional file 1: Table S9). An analysis of the synteny among the radish, *A. thaliana*, *B. rapa*, and *B. oleracea* genomes revealed the complete NBS-LRR genes were more syntenic than the partial genes.

**Fig. 6:**
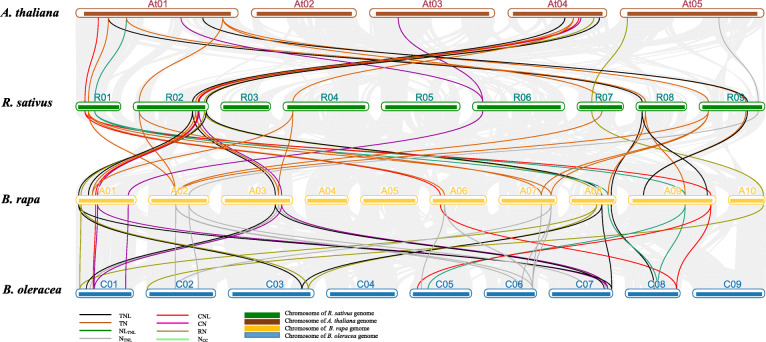
Synteny of 22 NBS-encoding genes between radish and *A. thaliana*, *B. rapa*, and *B. oleracea*. Gray lines in the background indicate the collinear blocks within the radish and other genomes. Different colored lines represent the relationships among orthologous gene pairs among the plant species

Finally, a comparative analysis of the orthologous pairs of NBS-LRR genes among four species (*R. sativus*, *A. thaliana*, *B. rapa*, and *B. oleracea*) revealed 22 *R. sativus* NBS-encoding genes with corresponding copies in the *A. thaliana*, *B. rapa*, and *B. oleracea* genomes (Fig. 6 and Additional file 1: Table S10). There was a one-to-one relationship among the NBS-encoding genes between the *R. sativus* and *A. thaliana* genomes. Additionally, half of the radish genes had single-copy syntenic genes in the *B. rapa* and *B. oleracea* genomes*.* Furthermore, the *B. rapa* and *B. oleracea* genomes had two and three copies of *RsTN38*, respectively, as well as three copies of *RsTN01*. Accordingly, these genes may have been important for the evolution of the NBS-LRR gene family.

The Ka/Ks ratio indicates the selective pressure on genes during evolution. We examined the Ka/Ks ratio for the orthologous gene pairs to determine the evolutionary selection patterns of NBS-LRR genes among *R. sativus*, *A. thaliana*, *B. rapa*, and *B. oleracea*. The Ka/Ks ratio reflects the number of non-synonymous substitutions per non-synonymous site (Ka) and the number of synonymous substitutions per synonymous site (Ks). The ratios for the orthologous gene pairs were estimated for each branch of the phylogenetic tree using the KaKs Calculator. The segmentally and tandemly duplicated NBS-LRR gene pairs as well as all orthologous NBS-LRR gene pairs had a Ka/Ks ratio < 1 (Additional file 1: Table S11), suggesting that the radish NBS-LRR gene family might have experienced strong purifying selection pressure during evolution.

### Radish NBS-LRR gene expression profiles in response to *F. oxysporum* f. sp. *raphanin 59* (*FOR59*)

**Fig. 7:**
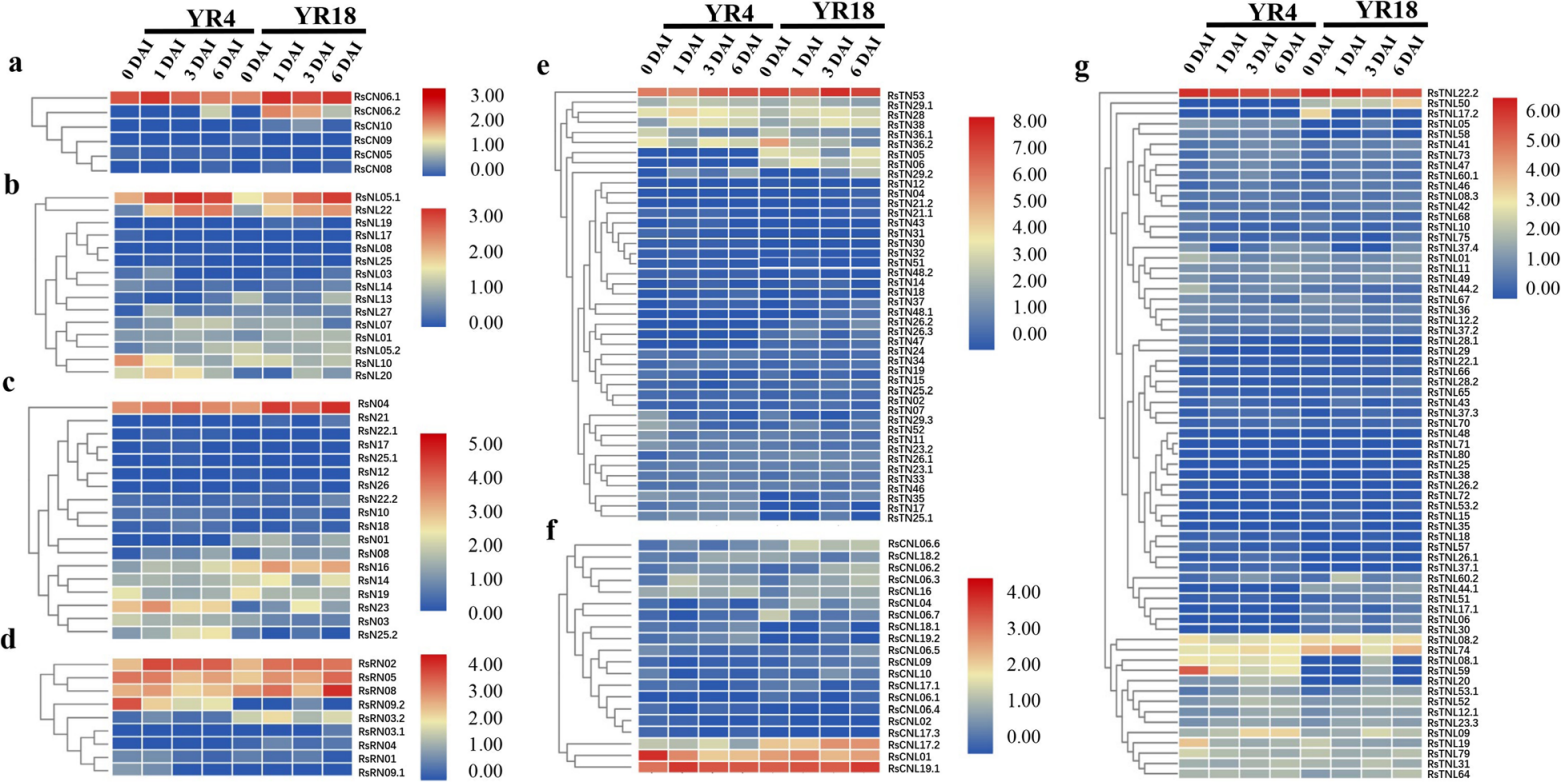
Expression profiles of NBS-LRR genes in whole seeding plant of radish. The ‘YR4’ and ‘YR18’ plants are resistant and susceptible plant lines, respectively. **a** Expression of *RsCN* genes. **b** Expression of *RsNL* genes. **c** Expression of *RsN* genes. **d** Expression of *RsRN* genes. **e** Expression of *RsTN* genes. **f** Expression of *RsCNL* genes. **g** Expression of *RsTNL* genes. DAI: days after inoculation

To identify NBS-LRR genes responsive to a *FOR59* infection and determine their spatiotemporal expression patterns, we analyzed the transcriptome data for all NBS-LRR genes. The transcriptome data were generated for whole plant seedlings of the ‘YR4’ and ‘YR18’ genotypes infected with *FOR59*; the whole seeding of ‘YR4’ and ‘YR18’ were collected on days 0, 1, 3, and 6 after the inoculation of *F. oxysporum* for the subsequent sequencing on the Illumina platform. Furthermore, we extracted the NBS-LRR genes from the generated RNA-seq data. Transcriptome data were obtained for 171 of 225 NBS-encoding genes based on the conserved domains and gene IDs. The remaining unidentified genes may be unexpressed in response to a *FOR59* infection. Among these 171 NBS-encoding genes, 29 were not differentially expressed between ‘YR4’ and ‘YR18’, and were minimally expressed at all time intervals. Thus, these genes were excluded from the gene expression analysis. The fragments per kilobase of exon per million fragments mapped (FPKM) values obtained for the 142 remaining NBS-LRR genes varied from 0 to 278.884. A total of 80 NBS-encoding genes had an FPKM value less than 1 FPKM (low expression), whereas there were 84 genes with more than 1 FPKM value, and less than 10 (high expression). Extremely high expression represented in 13 genes with more than 10, and less than 50; 1 gene with more than 50, less than 100; and 1 gene with more than 100, less than 300. The differentially expressed genes following the *FOR59* inoculation were analyzed with the R package edgeR [37], which revealed 36 genes that were more highly expressed in the resistant ‘YR4’ line than in the susceptible ‘YR18’ line at various time-points (Fig. 7). Seven genes (*RsCN10*, *RsN21*, *RsN26*, *RsNL19*, *RsTN04*, *RsTNL15*, and *RsTNL38*) were up-regulated only in ‘YR18’, whereas six genes (*RsCN05*, *RsCNL17.3*, *RsN22.1*, *RsTN31*, *RsTN32*, and *RsTN43*) were up-regulated in ‘YR4’. Most importantly, the expression of five genes (*RsN25.2*, *RsNL07*, *RsTN18*, *RsTNL09*, and *RsTN17*) gradually increased over time in the ‘YR4’ plants, but remained relatively stable in the ‘YR18’ plants, implying they may be crucial for the resistance to Fusarium wilt. However, the *RsTNL51* expression level was higher in ‘YR18’ than in ‘YR4’, and gradually increased during the infection period (Fig. 7g). These findings suggest a total of 75 NBS-LRR genes contribute to the resistance of radish to Fusarium wilt, with six genes (*RsN25.2*, *RsNL07*, *RsTN18*, *RsTNL09*, *RsTN17*, and *RsTNL51*) potentially crucial for the resistance.

### Responses to *FOR59* infections

**Fig. 8:**
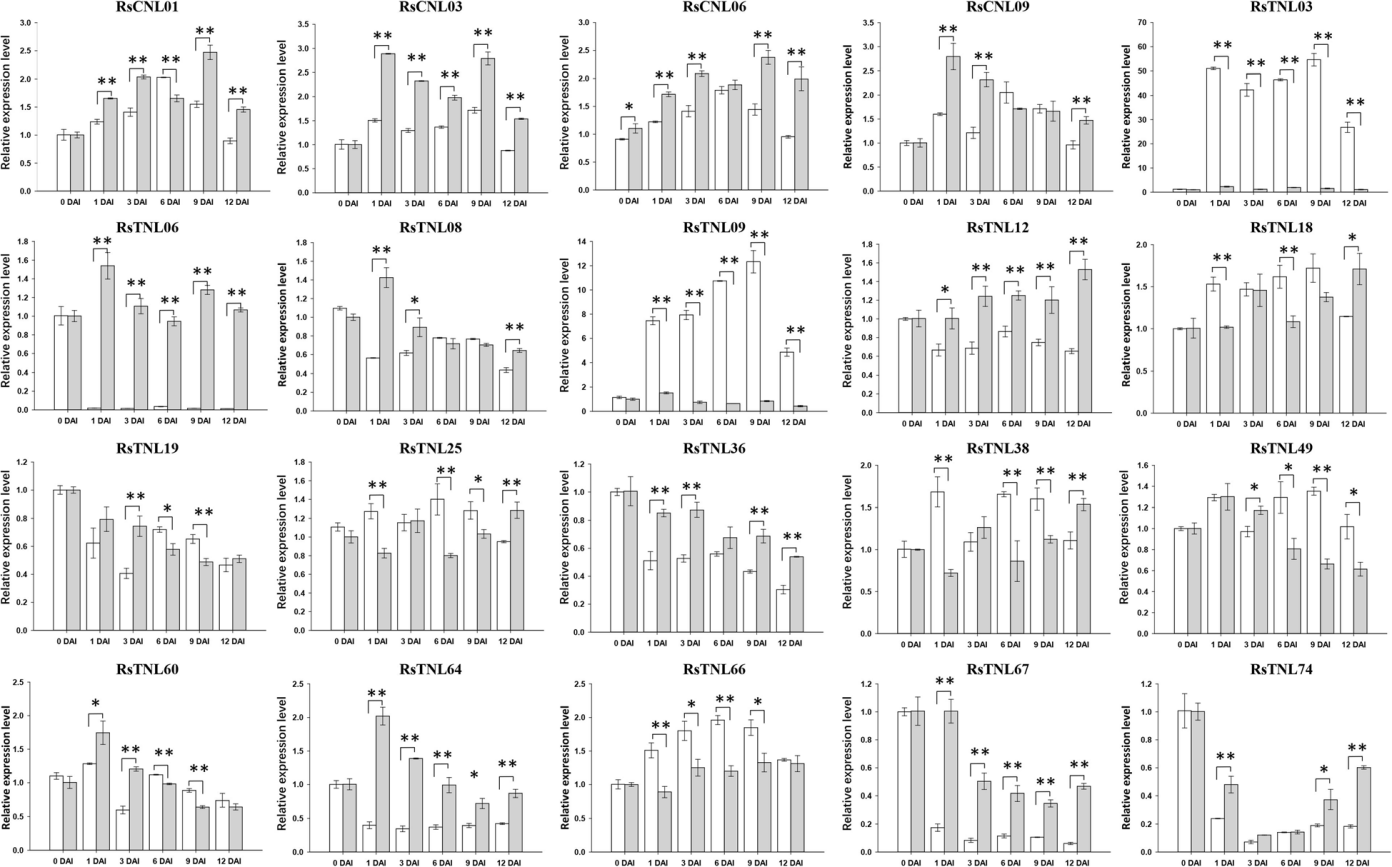
Relative expression levels of 4 *RsCNL* and 16 *RsTNL* radish genes. The ‘YR4’ (resistant) and ‘YR18’ (susceptible) lines are represented by white and gray bars, respectively. The y-axis represents the relative gene expression levels, whereas the time-points (0, 1, 3, 6, 9, and 12 DAI) are presented on the x-axis. The error bars indicate the standard deviation of three replicates; Asterisks indicate significant differences between the ‘YR4’ and ‘YR18’ lines based on a *t*-test (independent), ‘*’ indicates *P* value < 0.05 and > 0.01, and ‘**’ indicates *P* value < 0.01

The expression patterns of NBS-LRR-encoding genes in ‘YR4’ and ‘YR18’ plants were confirmed by a quantitative real-time (qRT)-PCR assay. Specifically, we validated the expression of only the CNL and TNL genes. Of the 19 *RsCNL* and 80 *RsTNL* genes in the radish genome, approximately 40 genes encoded proteins with amino acid differences between ‘YR4’ and ‘YR18’. The expression patterns of these genes in ‘YR4’ and ‘YR18’ at 0, 1, 3, 6, 9, and 12 days after inoculation (DAI) were determined, with the 18S rRNA gene used as an internal control. The *RsTNL03* (*Rs093020*) and *RsTNL09* (*Rs042580*) genes on chromosome R02 were significantly more highly expressed in the resistant ‘YR4’ plants than in the susceptible ‘YR18’ plants (Fig. 8), indicating these two genes are related to the Fusarium wilt resistance of radish. Interestingly, both genes were similarly expressed in ‘YR4’ and ‘YR18’ on day 0, but the infection rapidly up-regulated the expression levels in ‘YR4’ plants. The expression levels continued to increase until 9 DAI, further suggesting their potential role in Fusarium wilt resistance. Homologs of *RsTNL09* (*Rs042580*) were identified in *A. thaliana* (*AT4G36150*), *B. rapa* (*Bra010552* and *Bra011666*), and *B. oleracea* (*Bo3g154220* and *Bo7g117810*).

## Discussion

The NBS-LRR genes form a large family of stress resistance genes that are ubiquitous in all plant species. Genome-wide analyses of NBS-LRR gene families have been conducted for numerous species with sequenced genomes [17, 20, 38]. In the current study, we identified candidate NBS-LRR genes and studied their distribution, structure, clustering, duplication, synteny, and conservation. We identified 225 non-redundant NBS-encoding R gene candidates in the radish genome (Table 1, Additional file 1: Table S1) using highly stringent HMM and Pfam approaches. To confirm the accuracy of our method, a similar approach was used to identify NBS-encoding genes in *B. rapa*, *B. oleracea*, and *A. thaliana*. We identified 164, 212, and 244 NBS-encoding genes in *A. thaliana*, *B. rapa*, and *B. oleracea*, respectively, which is consistent with the results of an earlier investigation by Zhang et al. (2016), in which 165 and 204 NBS-encoding genes were detected in *A. thaliana* and *B. rapa*, respectively. Similar to our study, Yu et al. (2014) also identified 239 NBS-encoding genes in *B. oleracea*. However, the number of NBS-LRR genes identified in *A. thaliana*, *B. oleracea*, and *B. rapa* genomes differed in other studies [17, 18, 30]. These discrepancies are likely due to our stringent HMMER E-value as well as the manual checking for genes with partial NBS domains.

On the basis of a phylogenetic analysis of NBS-encoding candidate genes, 225 NBS-encoding genes were classified into the TNL and CNL subfamilies. The TNL subfamily included 80 TNL, 54 TN, 25 NL_TNL_, and 15 N_TIR_ genes, with the remaining 51 genes in the CNL subfamily comprising 19 CNL, 10 C, 9 RN, 2 NL_CC_, and 11 N_CC_ genes. Genes encoding RPW8-NBS-LRR proteins were not detected in radish. The CNL:TNL gene ratio was approximately 1:4, which is similar to that in *A. thaliana*, *B. oleracea*, and *B. rapa* [17]. This distinct pattern of TNL gene abundance suggests that the TIR domain is more functionally active than the CC domain in *Brassicaceae* species [39].

The NBS-LRR genes were unevenly distributed across the radish genome, with chromosomes R09 and R03 having the most and fewest genes, respectively. Additionally, 23 of the genes were located on diverse scaffolds (Fig. 2). Similar to other species, the NBS-LRR genes in radish mainly exist in clusters because of their rapid evolution [40, 41]. About 72% of the radish NBS-LRR genes were detected in 48 clusters, which is higher than the corresponding percentages in *A. thaliana* (61.7%), *B. rapa* (59.4%), and *B. oleracea* (60.3%) [17]. Moreover, 11 genes were included in cluster 44 of the radish genome. Gene families may expand because of polyploidizations, and tandem and local duplications are the most commonly evaluated mechanisms underlying gene family expansions [42]. We identified 66 NBS-encoding genes (29.33%) in radish that underwent tandem and segmental duplications, which is lower than the duplication rates for *A. thaliana* (55.7%), *B. rapa* (47.1%), and *B. oleracea* (43.3%) [17]. The TN genes were predominantly present in tandem arrays, and only three pairs of CN genes were detected in tandem arrays 2 (*RsCn02* and *RsCN03*), 4 (*RsCN07* and *RsCN08*), and 8 (*RsCNL11* and *RsCNL12*). Interestingly, *RsCNL14* and *RsTNL59*, which belong to two distinct subgroups (CNL and TNL), had undergone segmental duplications. These two genes were located in cluster 40 of the radish genome. We speculate that the NBS-LRR genes (especially TNL subfamily genes) in the radish genome had undergone inter- and intraspecific replications. Because radish and *A. thaliana* are *Brassicaceae* species, we investigated the crucifer blocks in the radish genome containing the identified NBS-LRR genes. A total of 45 NBS-LRR genes were located in the U block distributed on different chromosomes (R02, R04, and R08). The genes were also relatively abundant in the R (24 genes), D (23 genes), E (23 genes), and F (17 genes) blocks (Fig. 2). These observations suggest that the *R. sativus* genome structure arose following the rearrangement and divergence from a common ancestor with *A. thaliana* [43, 44]*.* All tandemly duplicated genes were found in the same clusters (i.e., highly similar), whereas most of the segmentally duplicated genes did not form clusters and were located on different chromosomes. The relatively few tandem and segmental duplications of NBS-encoding genes may help to explain why radish has evolved more slowly than other Brassicaceae species [45].

Gene duplication is considered as a major force for evolution [46]. The frequent sequence variation that can occur in the duplication process, can lead to domain’s structural variation, directly involved in protein functional role [47, 48]. In addition to this expansion, variation leads to neofunctionalization in family members. The neofunctionalization has been reported in KCS gene family and MADS box gene of *Arabidopsis* [49, 50]. According to the genome comparison in Brassica family members, we identified that the structural variation happened in many NBS-encoding genes which influenced the domain structure (Additional file 1: Table S12). For example, RsNL01 has the homolog gene in *A. thaliana, B. rapa,* and *B. oleracea*, but the number of exons was reduced, additionally LRR domain was added and TIR domain was lost in the *R. sativus*. Taken together of this results, we possibly suggest that these variation in the NBS-encoding genes might result in neofunctionalization or loss of function during the radish evolution.

The conserved structural domains of the radish TNL and CNL proteins were examined in this study. The NBS-LRR genes encoding the TIR domain were more common than the genes encoding the CC domain in the analyzed species (*R. sativus*, *A. thaliana*, *B. oleracea*, and *B. rapa*). However, the functions of several partial NBS-encoding genes lacking one or two domains (TIR, CC, and LRR) remain unknown, but their presence in various plant species imply they are important [17]. In the radish genome, the average number of exons was higher for TNL genes than for CNL genes, with half of the CNL genes containing only one exon (Fig. 4). This difference between gene types may be due to the conservative nature of CNL gene replications, which involve many regulatory components. This result is also consistent with those reported for *A. thaliana*, *B. rapa*, and *B. oleracea* [17, 20, 38]. Although the TNL and CNL genes are related to signaling and resistance specificity during pathogen recognition, the genes in these two subfamilies vary regarding sequences and associated signaling pathways and they cluster separately according to phylogenetic analyses [51]. Previous studies proved that the TNL group forms four phylogenetic clades in *B. rapa* [18] and *B. oleracea* [38]. Our motif analysis with the MEME program uncovered diverse motif compositions in the different RsTNL subfamilies. For example, the RsTNL-4 subgroup members had lost motifs 11, 12, and 16, whereas these motifs are present in the RsTNL-1 subgroup members. These differences may contribute to the functional divergence among the TNL proteins in radish.

Radish is an agronomically important root vegetable crop. Its genome, which contains triplicated segments, has intermediate characteristics between the *Brassica* A/C and B genomes, suggesting radish originated from a *Brassica* species [34]. According to a comparison with the *Brassica* A (Br), B (Bn), and C (Bo) genomes, the radish genome has been positioned between the *Brassica* A/C and B genomes [34]. Thus, an analysis of the synteny among the NBS-LRR genes of radish, *A. thaliana*, *B. rapa*, and *B. oleracea* may lead to novel insights into the evolutionary characteristics of RsNBS-encoding genes as well as the phylogenetic relationships with the genes in the other three species. In the current study, 39, 73, and 97 orthologous pairs were respectively identified between radish and *A. thaliana*, *B. rapa*, and *B. oleracea* (Fig. 5), implying that *R. sativus* is more closely related to *B. oleracea* than to *A. thaliana* or *B. rapa.*

Moreover, we identified genes in the *A. thaliana*, *B. rapa*, and *B. oleracea* genomes that correspond to 22 *R. sativus* NBS-encoding genes (Fig. 6). Additionally, the orthologous gene pairs may have existed before the ancestral divergence, with important roles related to the evolution of the NBS-LRR gene family [52]. Furthermore, we compared the Ka/Ks values of the orthologous gene pairs between *R. sativus* and *A. thaliana*, *B. rapa*, and *B. oleracea* lineages. For the CNL and TNL gene types, there were no significant differences in the orthologous gene pairs among the three species (Additional file 1: Table S11). We speculate that the NBS-LRR genes in *R. sativus*, *A. thaliana*, *B. rapa*, and *B. oleracea* may have been exposed to diverse selection pressures to develop genetic resistance to the same pathogen. Additionally, some pathogens may be specific to certain *Brassica* species.

In a previous study regarding radish resistance to Fusarium wilt, we isolated 68 NBS-RGAs and 46 SRLK-RGAs from two Fusarium wilt-resistant radish inbred lines, and the genetic diversity was analyzed with RGA-specific primers [53]. We also identified a major QTL (*Fwr1*) containing the *ORF4* gene, encoding a serine/arginine-rich protein kinase that may mediate Fusarium wilt resistance [54]. To further explore the mechanism underlying Fusarium wilt resistance, we profiled the expression of NBS-LRR genes by screening transcriptome data for the resistant ‘YR4’ and susceptible ‘YR18’ lines at different time-points during an infection (Fig. 7). A total of 75 NBS-LRR genes were identified as likely involved in the Fusarium wilt resistance of the ‘YR4’ line, including *RsNL07*, *RsTNL09* (chromosome R02), *RsTN17* and *RsTN18* (chromosome R04), and RsN25 (scaffold), which exhibited gradually up-regulated expression in infected ‘YR4’ plants. Accordingly, the genes belonging to the same cluster were similarly expressed. The identified genes and expression profiles based on transcriptome data were verified by a qRT-PCR analysis of the ‘YR4’ and ‘YR18’ lines at several time-points after plants were inoculated (Fig. 8). Of the analyzed genes, *RsTNL03* and *RsTNL09* were more highly expressed in ‘YR4’ (resistant) than in ‘YR18’ (susceptible) at different time-points. The *RsTNL09* (*Rs042580*) gene is homologous to *AT4G36150* in *A. thaliana*, *Bra010552* and *Bra011666* in *B. rapa*, and *Bo3g154220* and *Bo7g117810* in *B. oleracea*. The *Bra010552* gene encodes a TNL protein, and is one of the candidate genes for clubroot resistance in *B. rapa* [55]. The *RsTNL03* gene, which is a homolog of *AT4G16890* in *A. thaliana*, is likely involved in salicylic acid-dependent early plant defense responses [38–40]. Our RNA-seq and qRT-PCR data also indicated that *RsTNL06* (*Rs053740*) was up regulated in ‘YR18’ than in ‘YR4’ after the *FOR59* infection, taken together with the morphological characteristics (Additional file 1: Table S13) of ‘YR18’ (severe infected) suggests that RsTNL06 might be potential negative regulators of Fusarium wilt resistance in radish. Interestingly, *RsTNL06* was identified as an ortholog of the *B. oleracea* gene *Bo7g106630*, and may contribute to plant responses to *Fusarium* species [38]. The potential positive and negative regulators of Fusarium wilt resistance that were identified following the comprehensive analyses of RNA-seq and qRT-PCR data will need to be thoroughly functionally characterized to assess their utility for enhancing the Fusarium wilt resistance of radish.

## Conclusions

The NBS-LRR genes are important for the resistance of radish plants to various pathogens. A comprehensive analysis of the radish genome revealed 225 NBS-encoding genes, including 99 NBS-LRR genes and 126 partial NBS genes. Additionally, of the 202 NBS-encoding genes mapped onto nine chromosomes, 72% were located in clusters. Another 23 genes were located on different scaffolds. An analysis of syntenic and phylogenetic relationships among the NBS-LRR genes from *A. thaliana*, *B. rapa*, and *B. oleracea* provided valuable insights into the evolutionary characteristics of radish NBS-LRR genes. Moreover, 99 complete NBS-LRR genes were grouped in two main families (TNL and CNL), with the TNL genes further divided into four subgroups with highly similar exon-intron structures and motif compositions. In this study, we identified 75 NBS-LRR genes that protect radish plants from Fusarium wilt based on the transcriptome data. We also determined that *RsTNL03* (*Rs093020*) and *RsTNL09* (*Rs042580*) positively associated with the resistance to *F. oxysporum*, whereas *RsTNL06* (*Rs053740*) negatively associated with Fusarium wilt resistance in radish. The phylogenetic and gene expression data presented herein may help to clarify NBS-LRR gene functions.

## Methods

### Identification of NBS-encoding genes

To identify the NBS-encoding genes, we downloaded the whole-genome sequence from the radish database (http://radish-genome.org/). We applied hmmsearch of the HMMER (version 3.3) program [56] based on the HMM corresponding to the Pfam NBS (NB-ARC) domain (PF00931) to screen and identify the NBS-encoding genes in the radish genome. We also selected proteins with an E value <1e^− 20^ for sequence alignments with ClustalW [57]. We constructed the radish-specific NBS HMM with the hmmbuild module of HMMER (version 3.3) to rescan the radish protein database, and proteins with an E-value less than 0.01 were selected for further analyses [17, 58].

The N-terminal of NBS-containing proteins usually include the TIR and CC domains, whereas the C-terminal contains the RPW8 and LRR domains. We used the CLC main workbench (version 7.9) [59] and the Pfam (version 32) program [60] to detect domains in the NBS-containing proteins. These results were confirmed with the NCBI Conserved Domains Tool [61] and MEME (Multiple Expression motifs for Motif Elicitation) [62]. The CC domain in the protein sequences was identified with Paircoil2 [63], with a P score cut-off of 0.025. The NBS-encoding genes in the genomes of *A. thaliana* (Araport11), *B. rapa* (version 1.5), and *B. oleracea* (version 1.1) were also identified using the same method. The *A. thaliana* genome was download from the TAIR database (www.arabidopsis.org), whereas the *B. rapa* and *B. oleracea* genomes were downloaded from the database on the BRAD website (http://brassicadb.org/brad/).

### Multiple sequence alignment and phylogenetic analysis of NBS-encoding genes

These analyses confirmed the segregation between the two major NBS-encoding subpopulations (TNL and CNL) in the radish genome and clarified the phylogenetic relationships among the genes on the major branches. Multiple full NBS-containing protein sequences were aligned with the MUSCLE [64] program. The MEGA-X [65] program was used for the phylogenetic analysis, which was completed with the maximum likelihood method based on the Whelan and Goldman model [66]. Finally, the maximum likelihood method involving the LG model with 500 bootstrap replicates was used to construct the phylogenetic tree. Two additional phylogenetic trees were constructed with the same method. One tree was used for analyzing the relationships between the CNL and TNL genes in radish, whereas the other trees were used to verify that the radish CNL and TNL genes are consistent with the corresponding *A. thaliana* genes*.*

### Chromosomal locations, structures, and duplication events of NBS-encoding genes

The Mapchart (version 2.2) software was used to map all identified NBS-encoding genes onto *R. sativus* chromosomes based on their physical positions indicated in the *R. sativus* genome database. Several genes were organized in diverse NBS-LRR clusters, in which at least two NBS-encoding genes were localized in a 200-kb region and were separated by a maximum of eight non-NBS-LRR genes [35].

The Pepstats program (http://www.ebi.ac.uk/Tools/seqstats/emboss_pepstats) was used to calculate the pI and MW of the NBS-containing proteins. The promoter sequence (2000 bp upstream of the start codon) of each gene was examined with PlantCARE (http://bioinformatics.psb.ugen.be/webtools/plantcare/html/) to identify cis-elements [67].

The tandem and segmental duplications of the NBS-encoding genes in the radish genome were analyzed with the default parameters of MCScanX [68] and TBtools [69]. Gene duplications were confirmed based on the following two criteria: (a) the length of the shorter aligned sequence covered > 70% of the longer sequence; (b) the similarity of the two aligned sequences was > 70% [70]. The non-synonymous (Ka) and synonymous (Ks) substitutions for each duplication event were calculated with the KaKs Calculator (version 2.0) [71].

The predicted TNL and CNL proteins were analyzed with MEME [72], with default iterative cycles and the maximum number of motifs set to 20. Additionally, TBtools was used to visualize the structures of the TNL and CNL genes according to the genomic and coding sequences.

### Analysis of the orthologous gene pairs between *R. sativus* and three Brassicaceae species

Orthologous genes are important for investigating the evolutionary associations among diverse species. In this study, we identified the orthologous gene pairs between the *R. sativus* and *A. thaliana*, *B. rapa*, and *B. oleracea* genomes with the MCScanX program. Specifically, the following parameters were used: e = 1e^− 20^, u = 1, and s = 5 [73]. The orthologous pairs of NBS-encoding genes were extracted and the maps for analyzing synteny were prepared with TBtools. The 24 genome building blocks from the ancestral karyotype were assigned to the radish genome as previously described [34].

### Plant growth and *Fusarium oxysporum* inoculation

The two radish plant materials, inbred lines ‘YR4’ and ‘YR18’, were kindly provided by Seungho Kim (Neo Seed Co., Anseong, Republic of Korea). ‘YR4’ and ‘YR18’ are highly resistant and susceptible to *F. oxysporum*, respectively. To do the sampling, pathogen inoculation was used the modified root-dipping methods following the method by Baik et al. [74]. *F. oxysporum* f. sp. *raphanin* 59 was supported from the Korea Research Institute of Chemical Technology (Daejeon, Korea). Ten-day-old seedling of germplasm were grown at control condition at 25 °C and further were subjected to water rinse to remove the soil from the root, and inoculated in spore suspension of *F. oxysporum* with concentration of conidia. 3 × 10^6^ mL^− 1^ for 30 min. Later, all individual seedlings were transplanted into 50-well seedling tray containing autoclaved soil. After inoculation, all plants were incubated in the controlled chamber, 80% humidity and 25 °C for 1 day without light. Further, these plants were grown in the controlled chamber with 60% humidity and 25 °C with 12 h/12 h condition. Individual seedlings of ‘YR4’ and ‘YR18’ were collected at 0, 1, 3, 6, 9, and 12 days after inoculation (DAI). Three individuals were used for subsequent RNA isolation and qRT-PCR analysis.

### Total RNA extraction and gene expression analysis

Total RNA was isolated from the ‘YR4’ and ‘YR18’ plants with the RNeasy Mini Kit (QIAGEN, Germany). The RNA quality and quantity were investigated through agarose gel electrophoresis and with the Nanodrop 2000 spectrophotometer (Thermo Scientific, USA). The purified total RNA (2 μg) was reverse transcribed to cDNA with the oligo-(dT) primer and TOPscript™ reverse transcriptase (Enzynomics Company, Korea). A qRT-PCR assay was performed with the SYBR Green Supermix and the CFX96™ Real-Time System (Bio-Rad Company, USA) using subsequent conditions: 95 °C for 3 min; 39 cycles of 95 °C for 15 s and 58 °C for 20 s. Relative gene expression levels were calculated as per the 2^−∆∆Ct^ method [75].

Previously generated transcriptome data for the *FOR59*-inoculated ‘YR4’ and ‘YR18’ lines at four time-points (0, 1, 3, and 6 DAI) were analyzed (Accession number PRJNA643982) (Additional file 1: Table S14). The growth conditions of plant materials were mentioned earlier and two biological replications were used for RNA-Seq. The brief summary of RNA sequencing and library preparation is as follows. Firstly rRNA was removed from total RNA using RIBO COP rRNA depletion kit (LEXOGEN, Inc., Austria). Total RNA was used to prepare cDNA libraries using the NEBNext Ultra II Directional RNA-Seq Kit (NEW ENGLAND BioLabs, Inc., UK) as per manufacture’s instruction. Then, libraries were examined with the Agilent 2100 bioanalyzer (DNA High Sensitivity Kit) to estimate the fragment size and quantified with the library quantification kit via a StepOne Real-Time PCR System (Life Technologies, USA). High-throughput paired-end sequencing was carried out using Hiseq X10 (Illumina, Inc., USA). Total RNA-Seq reads were mapped using TopHat software tool [76] to get bam file (alignment file). These alignment files were used to assemble transcripts, estimate their abundances and detects differential expression of genes using cufflinks. We used the FPKM (fragments per kilobase of exon per million fragments) method for determining the expression level of the gene regions. The FPKM data were normalized based on Quantile normalization method using EdgeR within R [37]. Additionally, heat maps were produced with TBtools.

## Supplementary Information

**Additional file 1: Table S1.** Information regarding the radish NBS-LRR genes. **Table S2.** Clusters of radish NBS-LRR genes. **Table S3** Predicted promoter elements of NBS-encoding genes in the radish genome. **Table S4.**Analysis and distribution of conserved motifs in radish CNL and TNL proteins. **Table S5.** MEME analysis of the CNL and TNL proteins. **Table S6.** Tandem arrays of the radish NBS-LRR genes. **Table S7.** Segmentally duplicated radish NBS-LRR genes. **Table S8.** Ka/Ks values for the segmentally duplicated genes in the radish genome. **Table S9.** Segmentally duplicated NBS-LRR genes in the radish*/A. thaliana*, radish/*B. oleracea*, and radish/*B. rapa* genomes. **Table S10.** Common segmentally duplicated NBS-LRR genes in the radish*/A. thaliana*, radish/*B. oleracea*, and radish/*B. rapa* genomes. **Table S11.** Ka/Ks values between radish and three related plant species. **Table S12.** The information for gene structure and domain change of radish/*A. thaliana*, radish/*B. oleracea*, and radish/*B. rapa* genomes. **Table S13.** The resistance of radish lines to *F. oxysporum f. sp. Raphani* 59. **Table S14.** The summary of the RNA-Seq database. **Table S15.** Details regarding the qRT-PCR primers. **Table S16.** The transcriptome data of the radish lines after inoculated with *F. oxysporum f. sp. Raphani 59 (XLSX 15325 kb)*

**Additional file 2: Figure S1.** Distribution of TNL, CNL, and partial genes. Distributions for each class are presented across the radish chromosomes. RUS, scaffold summary. Bars are divided into CNL genes (orange), TNL genes (blue), and partial genes (green)

**Additional file 3: Figure S2.** Phylogenetic tree representing the relationships of  NBS-encoding genes between radish and arabidopsis. Different colored arcs represent the different groups (or subgroups) of NBS-encoding genes.

**Additional file 4: Figure S3.** Relative expression levels of *RsCNL* and *RsTNL* radish genes. The ‘YR4’ (resistant) and ‘YR18’ (susceptible) lines are represented by white and gray bars, respectively. The y-axis represents the relative gene expression levels, whereas the time-points (0, 1, 3, 6, 9, and 12 DAI) are presented on the x-axis.

## Data Availability

All datasets generated and analyzed during this study are included in this article and its additional files. The RNA-seq data generated during the current study have been deposited and publicly available at the National Center of Biotechnology Information (NCBI, http://www.ncbi.nlm.nih.gov/) repository under the accession number PRJNA643982.

## Abbreviations

CC: Coiled-coil
CNL: Coiled-coil nucleotide-binding site–leucine-rich repeat
*FOR*: *Fusarium oxysporum* f. sp. *raphanin 59*
FPKM: Fragments per kilobase of exon per million fragments mapped
HMM: Hidden Markov model
NBS-LRR: Nucleotide-binding site–leucine-rich repeat
RPW8: Resistance to powdery mildew 8
TNL: Toll/interleukin 1 receptor nucleotide-binding site–leucine-rich repeat
