# Supplementary figures and images for: Genome-wide identification and characterization of NBS-encoding genes in Raphanus sativus L. and their roles related to Fusarium oxysporum resistance
Source: BMC Plant Biol. 2021 Jan 18;21:47. doi: 10.1186/s12870-020-02803-8 (PMC7814608; doi:10.1186/s12870-020-02803-8)

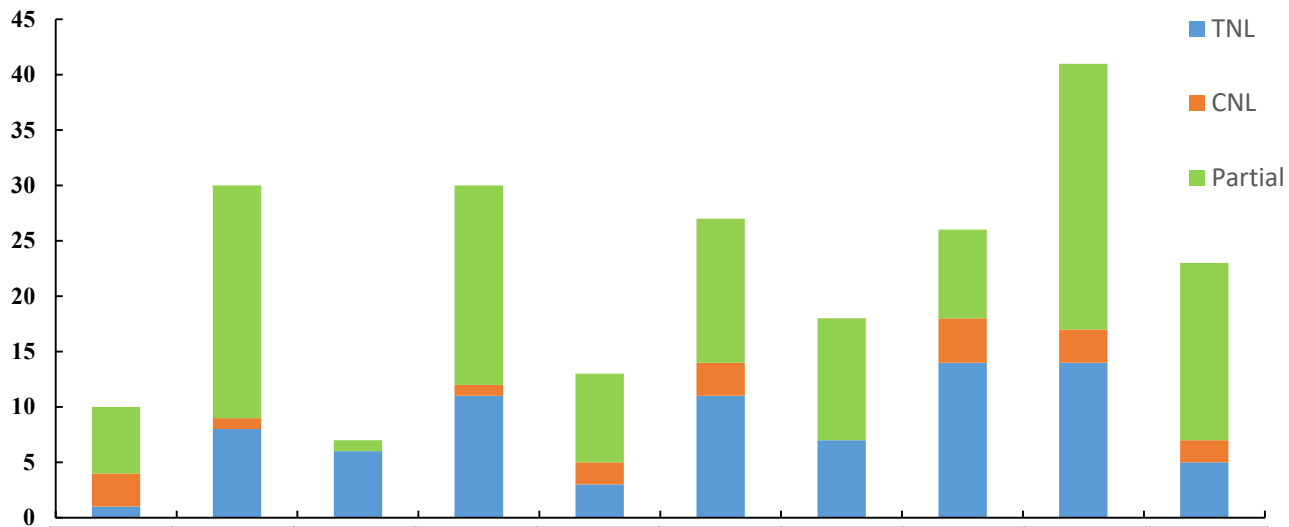

|         | R01 | R02 | R03 | R04 | R05 | R06 | R07 | R08 | R09 | RUS |
|---------|-----|-----|-----|-----|-----|-----|-----|-----|-----|-----|
| TNL     | 1   | 8   | 6   | 11  | 3   | 11  | 7   | 14  | 14  | 5   |
| CNL     | 3   | 1   | 0   | 1   | 2   | 3   | 0   | 4   | 3   | 2   |
| Partial | 6   | 21  | 1   | 18  | 8   | 13  | 11  | 8   | 24  | 16  |

Supplement: Supplementary file 2 — Additional file 2: Figure S1. Distribution of TNL, CNL, and partial genes. Distributions for each class are presented across the radish chromosomes. RUS, scaffold summary. Bars are divided into CNL genes (orange), TNL genes (blue), and partial genes (green) [file 12870_2020_2803_MOESM2_ESM.pdf]

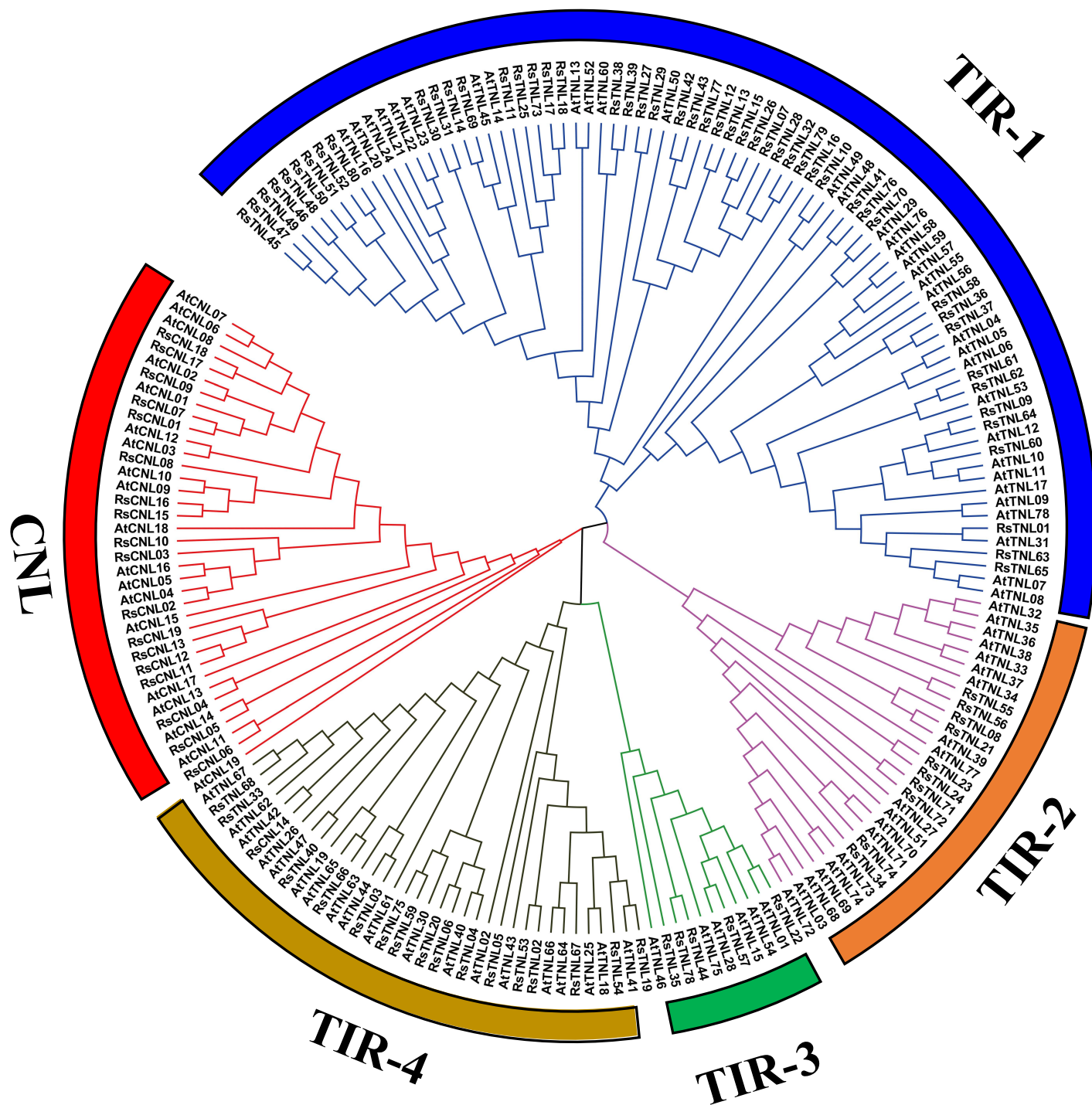

Supplement: Supplementary file 3 — Additional file 3: Figure S2. Phylogenetic tree representing the relationships of NBS-encoding genes between radish and arabidopsis. Different colored arcs represent the different groups (or subgroups) of NBS-encoding genes. [file 12870_2020_2803_MOESM3_ESM.pdf]

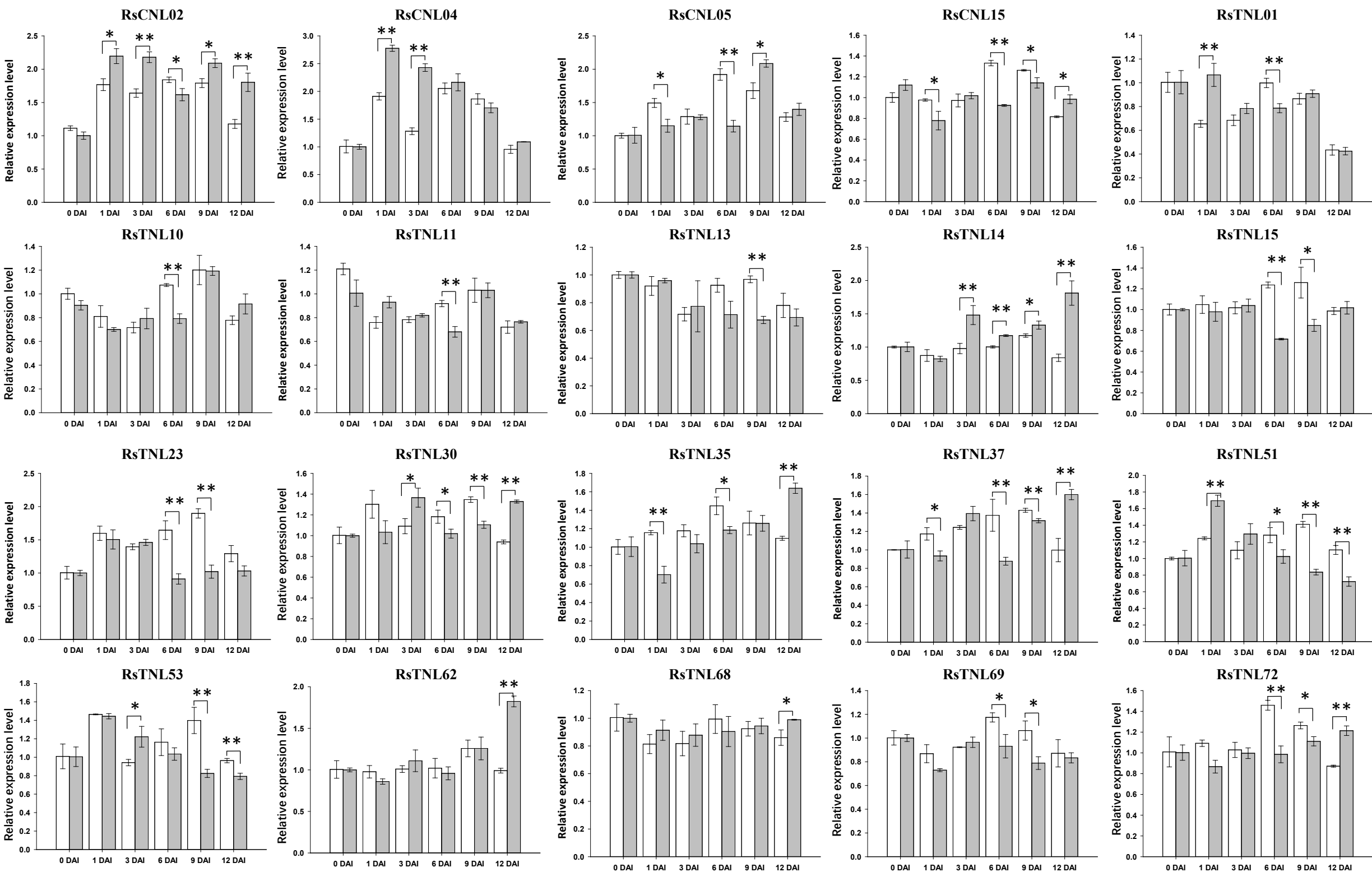

Supplement: Supplementary file 4 — Additional file 4: Figure S3. Relative expression levels of RsCNL and RsTNL radish genes. The ‘YR4’ (resistant) and ‘YR18’ (susceptible) lines are represented by white and gray bars, respectively. The y-axis represents the relative gene expression levels, whereas the time-points (0, 1, 3, 6, 9, and 12 DAI) are presented on the x-axis. [file 12870_2020_2803_MOESM4_ESM.pdf]
